# Effect of oral spray with *Lactobacillus* on growth performance, intestinal development and microflora population of ducklings

**DOI:** 10.5713/ajas.19.0052

**Published:** 2019-07-01

**Authors:** Qi Zhang, Yuchen Jie, Chuli Zhou, Leyun Wang, Liang Huang, Lin Yang, Yongwen Zhu

**Affiliations:** 1 Guangdong Provincial Key Laboratory of Animal Nutrition Control, College of Animal Science, South China Agricultural University, Guangzhou 510642, China

**Keywords:** Ducklings, Intestinal Development, *Lactobacillus*, Oral Spray

## Abstract

**Objective:**

The aim of this study is to investigate the effect of oral spray with probiotics on the intestinal development and microflora colonization of hatched ducklings.

**Methods:**

In Exp. 1, an one-way factorial design was used to study the antibacterial activity of the probiotics and metabolites on *Escherichia coli* (*E. coli*) without antimicrobial resistance. There were four experimental groups including saline as control and *Lactobacillus*, *Bacillus subtilis*, combined *Lactobacillus* and *Bacillus subtilis* groups. In Exp. 2, 64-day-old ducklings were allotted to 2 treatments with 4 replicated pens. Birds in the control group were fed a basal diet supplemented with *Lactobacillus* fermentation in the feed whereas birds in the oral spray group were fed the basal diet and administrated *Lactobacillus* fermentation by oral spray way during the first week.

**Results:**

In Exp. 1, the antibacterial activities of probiotics and metabolites on *E. coli* were determined by the diameter of inhibition zone in order: *Lactobacillus*>combined *Lactobacillus* and *Bacillus subtilis*>*Bacillus subtilis*. Additionally, compared to *E. coli* without resistance, *E. coli* with resistance showed a smaller diameter of inhibition zones. In Exp. 2, compared to control feeding group, oral spray group increased (p<0.05) the final body weight at d 21 and average daily gain for d 1–21 and the absolute weight of the jejunum, ileum and total intestine tract as well as cecum *Lactobacillus* amount at d 21.

**Conclusion:**

*Lactobacillus* exhibited a lower antibacterial activity on *E. coli* with resistance than *E. coli* without resistance. Oral spray with *Lactobacillus* fermentation during the first week of could improve the intestinal development, morphological structure, and microbial balance to promote growth performance of ducklings from hatch to 21 d of age.

## INTRODUCTION

The immediate post-hatch period is critical for the development of gastrointestinal tract to minimize the mortality and keep uniformity of young poultry [1]. During first post-hatched days the small intestine undergoes dramatic physiological and morphological changes to increase nutrient digestion and absorption [2,3]. For example, the weight and length of small intestine increases more rapidly than the whole body mass and reaches a maximum between 3 and 7 days [4]. In addition, in newly hatched birds a major change in the source of nutrients occurs by switching from yolk nutrition to enteral nutrition [5]. Early nutrient supply to young poultry is essential for improving the intestine growth and nutrient intake [6] via stimulating digestive enzymes secretion and increasing yolk sac nutrient utilization [7]. Therefore, it is necessary to explore whether the use of neonatal probiotics supplements could promote intestinal health early in life to maximize the nutrient efficiency in later life.

Using probiotics to improve the intestine health of poultry is not a new concept, how ever, a complete understanding of when and how to use probiotics still has great potential. The beneficial effects of probiotics served as alternative feed additives to antibiotics on improving the intestinal microbial balance, morphological structure and feed utilization have been proved in poultry production [8]. Therefore, it is important to select probiotics as supplements to aid in the proper development and microflora colonization of the intestinal tract in hatched birds as soon as possible [9]. Due to the dependence on the residual yolk nutrition and the lower feed intake of birds during the first few days post-hatch [10], it is speculated that provision of probiotics supplied by feeding might not be enough to exert some effect on the rapid development of intestine of birds in early life. Therefore, a new method of supplying probiotics using oral spray was adopted to obtain the greater intestinal health and nutrient efficiency in newly hatched birds in the present study. Firstly *in vitro*, the inhibitory effect of *Lactobacillus* and metabolites on *Escherichia coli* (*E. coli*) (antimicrobial resistance vs non-antimicrobial resistance) was investigated (Exp. 1). Secondly, *in vivo*, the effect of *Lactobacillus* fermentation in oral spray way during the first week of on growth performance, intestinal development, and gut flora amount was evaluated in ducklings from hatch to 21 d of age (Exp. 2).

## MATERIALS AND METHODS

All experimental procedures were approved by the Institutional Animal Care and Use Committee of South China Agricultural University (SCAU-AEC-2010-0416). This study included 2 experiments as follows: the antibacterial activity of probiotics and metabolites on *E. coli in vitro* (Exp. 1) and the effect of *Lactobacillus* fermentation in oral spray way during the first week on performance and intestinal development of ducklings from hatch to 21 d of age *in vivo* (Exp. 2).

### Microorganisms and medium

Avian *E. coli* ATCC25922 without antimicrobial resistance was purchased from China General Microbiological Culture Collection Center (Beijing, China), while avian *E. coli* ATCC 25922 with antimicrobial resistance was kindly provided by Professor Sun in College of Veterinary Medicine of South China Agricultural University (Guangzhou, China). *Lactobacillus* and *Bacillus subtilis* were isolated from a commercial product in our lab by conventional microbiological identification methods [11]. *E. coli*, *Lactobacillus* and *Bacillus subtilis* were cultured in mediums (Boyao Biotechnology Company, Shanghai, China) of Eosin-Methyl Blue Agar broth (#BS1041), MRS broth (#BS1138), and Nutrient Agar broth (#BS1002), respectively.

### Antimicrobial sensitivity test

Firstly, *Lactobacillus* and *Bacillus subtilis* were fermented in Luria broth (#BS2078) for 24 h in anaerobic and aerobic conditions to prepare the solutions of probiotics fermentation without filtration, respectively. In Exp. 1, an one-way factorial design was used to study the antibacterial activity of the probiotics and metabolites on *E. coli* without antimicrobial resistance. There were four experimental groups including saline as control and *Lactobacillus*, *Bacillus subtilis*, combined *Lactobacillus* and *Bacillus subtilis* groups. The combined mixture was prepared by an equal volume of *Lactobacillus* and *Bacillus subtilis* fermentations. Oxford cups were punched into the plates and were loaded with 2 mL probiotics culture stock solution. After overnight incubation at 37°C, the diameters of the inhibition zones were determined to examine the antibacterial activity of against test *E. coli* [12]. All procedures were performed in three replicates for three times. Then, the *Lactobacillus* fermentation group presenting a greater inhibitory effect on the *E. coli* was screened and selected to evaluate the antibacterial activity between two *E. coli* sources with and without antimicrobial resistance.

### Birds, sample collection and analyses

In Exp. 2, 64-day-old Cherry Valley ducklings were weighed individually and allotted to 2 treatments with 4 replicated pens of 8 ducklings per pen based on similar body weight (BW). All ducklings were reared on wire floors in an environmentally controlled room with adjustment of temperature and humidity from 0 to 21 d of age. Birds in the control group were fed a basal diet supplemented with probiotics fermentation in the feed whereas birds in the oral spray group were fed the basal diet and administrated probiotics fermentation by oral spray during the first week. To determine the antibacterial activity of probiotics and metabolites on *E. coli in vitro*, the screened *Lactobacillus* was fermented in 50 g/L brown sugar solution as prepared like in Exp. 1. For the oral spray group, 1 mL *Lactobacillus* fermentation (10^9^ colony forming units [CFU]/mL) were taken and supplied for each duckling by oral spraying at 1 d of age. Oral spray produces a spray of balanced tiny droplets of fermentation of *Lactobacillus* and delivers it directly into duckling’s mouth twice each day. The dose of liquid fermented *Lactobacillus* was increased step-wise by 1 mL/d for each duckling until 7 d of age. For the control group, ducklings were fed the basal diet supplemented with an equal amount of *Lactobacillus* fermentation to that used in the spray group per pen during the first week. From 8 to 21 d of age, all ducklings in the two groups were just fed the same basal diet without any *Lactobacillus* fermentation supplementation or oral spray. Feed intake was recorded each day per pen. The basal diet was formulated to meet or exceed the nutrient requirements recommended by NRC (1994) for ducklings at the starter period. Compositions and nutrient levels of the basal diet are presented in Table 1. Feed and water were provided *ad libitum* and no mortality of birds were observed throughout the experimental period. At d 7 and 21, after 12 h feed withdrawal, birds were weighed by each replicate pen. The average daily gain (ADG), average daily feed intake (ADFI), and feed:gain ratio (F:G) were calculated. At d 21, based on the average BW per pen, 2 birds in each pen were euthanized by CO_2_ inhalation, and the duodenum, jejunum, and ileum were separated for the measurements of weight and length. The relative weight and length of the duodenum, jejunum, and ileum were calculated based on the BW. Then, segments of about 1.5 cm from the middle of duodenum, jejunum, and ileum were excised and flushed with ice-cold saline and immediately placed in 4% paraformaldehyde for morphometric analysis. The indices of villus height, crypt depth and muscular thickness were measured using computer-aided light microscope image analysis as described by Uni et al [4]. The chyme in caecum of one duckling was selected for measuring the content of total colonies, *E. coli* and *Lactobacillus* by the plate CFU method [13].

### Statistical analyses

In Exp. 1, the data about the diameter of inhibition zones on *E. coli* without antimicrobial resistance were analyzed by one-way analysis of variance using the general linear model procedure of SAS 9.2 (SAS, 2009). Differences among means were tested by the least significant difference method. The data about the diameter of inhibition zones between two *E. coli* sources with and without antimicrobial resistance (Exp. 1) and growth performance, intestine weight and length, morphological structure, and cecal flora number of ducklings between the control and oral spray groups were analyzed by an independent samples t-test. Statistical significance was set at p<0.05.

## RESULTS

### Diameter of inhibition zone

**Figure 1:**
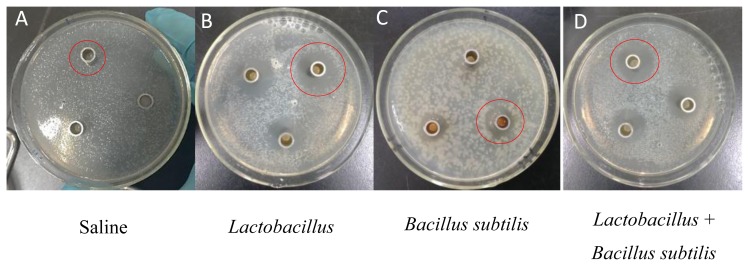
Representative inhibition zone of *Escherichia coli* (*E. coli*) without resistance. The diameters of the inhibition zones were determined to examine the antibacterial activity of probiotics culture stock solution against test *E. coli* (see red circle). The order of antibacterial activity was that *Lactobacillus* (B)> combined *Lactobacillus* and *Bacillus subtilis* (D) > *Bacillus subtilis* (C) > saline (A).

**Figure 2:**
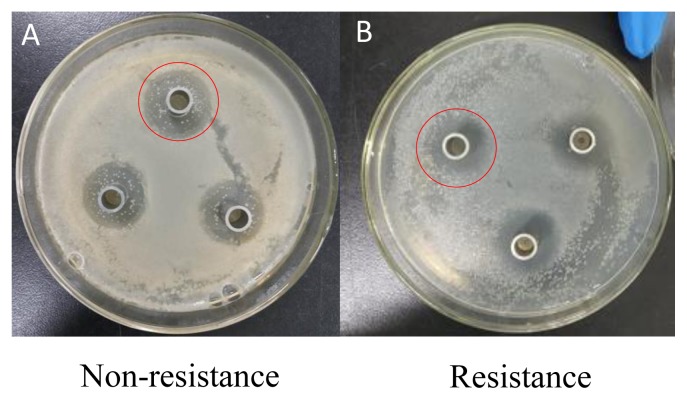
Representative inhibition zone of *Escherichia coli* (*E. coli*) with or without resistance. The *Lactobacillus* fermentation with a greater inhibitory effect on the *E. coli* was selected to evaluate the antibacterial activity between two *E. coli* sources with and without antimicrobial resistance (see red circle). The order of antibacterial activity was that non-resistance (A) > resistance (B).

In Exp. 1, the diameter of inhibition zones from *Lactobacillus* group was greater than that from *Bacillus subtilis* group or combined *Lactobacillus* and *Bacillus subtilis* group (p<0.01), while the diameter of inhibition zones from combined *Lactobacillus* and *Bacillus subtilis* group was greater than that from *Bacillus subtilis* group (p<0.01, Table 2; Figure 1). Under *Lactobacillus* fermentation treatment, *E. coli* with antimicrobial resistance showed a smaller diameter of inhibition zones compared to *E. coli* without antimicrobial resistance (p<0.05, Table 3; Figure 2).

### Growth performance

In Exp. 2, oral spray with *Lactobacillus* fermentation during the first week had no effect on the final BW at 21 d and ADG at d 1 to 7 as well as ADFI and F:G at d 1 to 7 and d 1 to 21 (p> 0.05; Table 4). Compared to the control group, oral spray group had an increased final BW at 21 d and ADG of birds at d 1 to 21(p<0.05; Table 4).

### Weight and length of intestine

The data of absolute weight and length of duodenum, jejunum, ileum and total intestine tract at d 21 are presented in Table 5. Oral spray group increased (p<0.05) the absolute weight of jejunum, ileum, and total intestine tract and did not influence other above-mentioned indices (p>0.05) of ducklings at d 21 compared to the control group.

### Intestinal histomorphology

**Figure 3:**
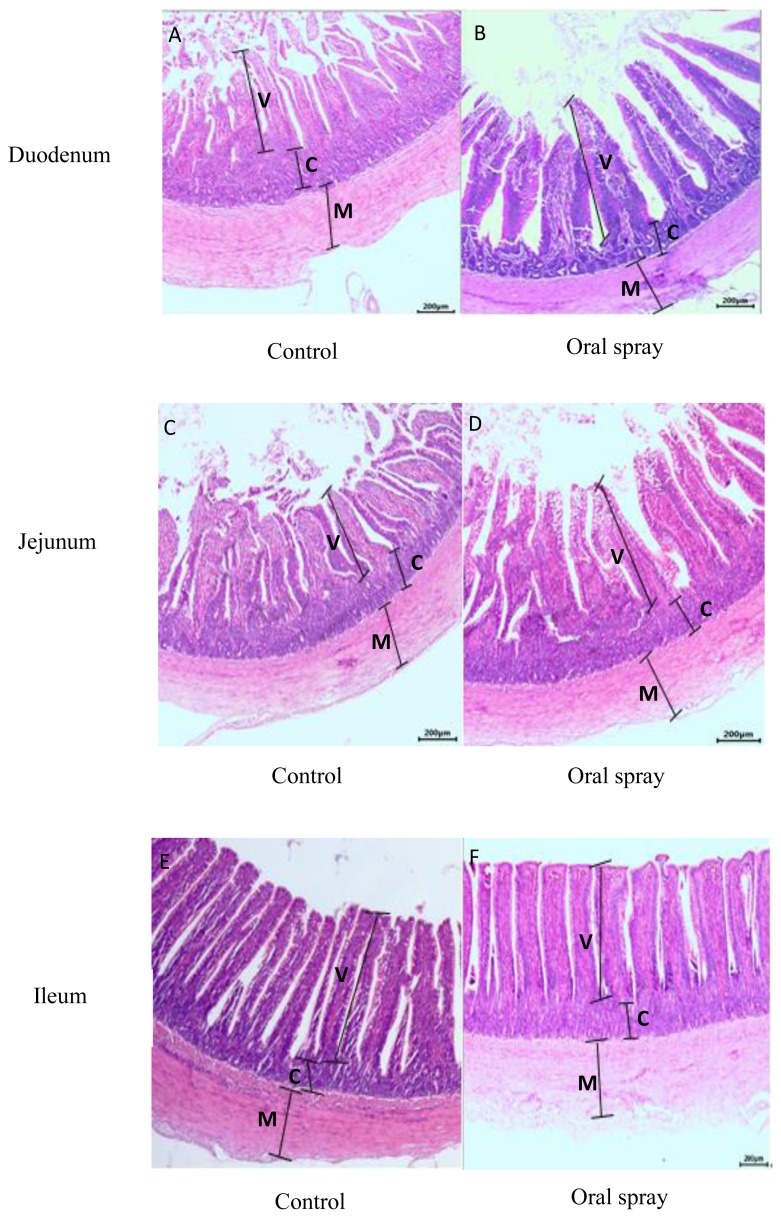
Representative light microscopy (×200) of the histomorphology of duodenum (A, B), jejunum (C, D) and ileum (E, F) of the ducklings at 21 of age. V, villus; C, crypt; M, muscularis mucosa; control group, the basal diet supplemented with *Lactobacillus* fermentation; oral spray group, ducklings fed the basal diet and administrated *Lactobacillus* fermentation by oral spray way.

The data and representative light microscopy of villus height, crypt depth, musculature thicknesses and villus height:crypt depth ratio of duodenum, jejunum and ileum are shown in Table 6, Figure 3, respectively. Oral spray with *Lactobacillus* fermentation during the first week increased (p<0.05) the villus height and villus height:crypt depth ratio of duodenum and jejunum and musculature thicknesses of jejunum as well as decreased the crypt depth of duodenum compared to the control group, but did not affect the intestinal histomorphology of ileum (p>0.05).

### Cecal microflora population

Compared with the control group, oral spray with *Lactobacillus* fermentation during the first week increased (p<0.05, Table 7) the cecal *Lactobacillus* amount and had no effect (p>0.05) on the amounts of *E. coli* and total colonies and *Lactobacillus*:*E. coli* ratio.

## DISCUSSION

The use of probiotics has become more common to achieve greater productivity and health benefits in the poultry production [8]. Numerous *in vivo* studies in broilers [14], turkeys [15], and layers [16] have proved that probiotics as feed additive can improve nutrient utilization, gut health, and immune function, resulting in better production performance, such as greater BW gain and resistance to infectious bacteria. However, several other workers reported that no beneficial effects were observed in birds given diets supplemented with or without probiotics [17,18]. Variations in the efficacy of probiotics may depend upon the stability and efficiency of the probiotics and species or strains of microorganisms given to the host [19]. Considering the susceptibility to *E. coli* infections in poultry at the post-hatched period, the inhibitory effect of probiotics against *E. coli* was investigated *in vitro* in the present study. According to the diameter of inhibition zone, the antibacterial activity of probiotics culture was *Lactobacillus* > combined *Lactobacillus* and *Bacillus subtilis* > *Bacillus subtilis*. As indicated previously [20], the manner by which *Lactobacillus* inhibits the growth and proliferation of pathogenic bacteria is by lowering the pH with the production of primary metabolites such as organic acids and hydrogen peroxide. The use of antibiotics in poultry feed as a growth promoter has been restricted in many countries around the world [21]. Probiotics are considered alternative microbial feed additives to enhance growth and disease prevention for birds by improving the intestinal microbial balance [22]. Therefore, in the present study the screened *Lactobacillus* were selected to evaluate the antibacterial activity between two *E. coli* sources with and without antimicrobial resistance as determined by the diameter of the inhibition zone. Compared to *E. coli* without resistance, *E. coli* with resistance displayed a smaller diameter of inhibition zones under *Lactobacillus* fermentation treatment, implying that *E. coli* with antimicrobial resistance exhibited a greater resistance of *Lactobacillus* fermentation. It is suggested that the possibility of replacing antibiotics in poultry production might not only depend on one alternative feed additive alone, such as probiotics, enzymes, and acidifiers etc, but requires a comprehensive nutrition strategy together with good breeding and management conditions [20].

The key point of adding beneficial bacteria to improve the productive performance and intestine health of poultry lines is in understanding completely when and how to use them. Some studies reported that there are positive effects on production efficiency of broiler chickens fed diets containing *Lactobacillus* cultures [14,23,24] or *Lactobacillus* fermentation administered intragastrically [25]. Other reporters found that no significant differences were observed in weight gain of chicken given diets with or without *Lactobacillus* cultures [17,18]. These inconsistent results may be due to the differences in the stability and specificity of the *Lactobacillus* strain to the host, exact dose and supply way of *Lactobacillus* as well as the developmental period and nutritional status of the birds. For example, in the newly hatched chick, the small intestinal development and function appears to be immature and should be further improved to minimize the mortality and keep uniformity of young poultry [4]. Therefore, it is important to select probiotics as supplements to aid in the proper development and microflora colonization of the intestinal tract in the early life of hatched birds as soon as possible [9]. In the current study, the beneficial effects of the *Lactobacillus* fermentation supplements supplied by oral spray were examined. Compared to the feeding control group, oral spray with *Lactobacillus* fermentation during the first week had no effect on growth performance of ducklings during 1 to 7 d of age while positively increased the final BW at 21 d and ADG from 1 to 21 d. Due to the dependence on the residual yolk nutrition and the lower feed intake of birds during the first few days post hatch, it was presumed that provision of probiotics supplied by the traditional feeding way might be insufficient to exert some effect on the rapid development of intestine of birds in early life and then could not maximize the value of the nutrient efficiency compared to oral spraying. In our study, the greater dose of *Lactobacillus* fermentation by oral spray at the critical post-hatch period might be more conducive to the proper development of the intestinal tract to obtain the greater digestion and utilization of nutrients in long run.

The interaction of intestinal growth, digestive functions, and enteral nutrition are critical for hatched poultry during the post-hatch period [4]. In order to increase digestion and absorption of nutrients from the exogenous feed, physiological and morphological changes of the small intestine are dramatic in birds at the first post-hatched days [2,3]. For example, the weight and length of the small intestine increased more rapidly than the whole body mass and reach a maximum between 3 and 7 days [3,4]. Therefore, early nutrient supply to young poultry is essential for improving the intestine growth and nutrient intake [6]. Access to supplements stimulating digestive enzyme and yolk sac nutrient utilization in early life can promote the intestinal development [7]. In the current study, ducklings given *Lactobacillus* fermentation immediately during the first week exhibited a greater absolute weight of the jejunum, ileum, and total intestine tract. Similar results about promoting intestine development were consistent with those reported in broilers fed probiotics [26]. Therefore, the sooner the gastrointestinal tract achieves functional capacity, the more nutrients can be utilized efficiently, leading to increased weight gain from d 1 to 21. In addition, the immediate post-hatch period is critical for intestinal morphological development in order to enlarge the intestinal absorptive surface and increase nutrient supply [3]. Thus, some of the enhanced growth effects of early nutrition may be explained by changes in intestinal tract development. Moreover, oral spray with *Lactobacillus* and metabolites during the first week increased the villus height and villus height:crypt depth ratio of duodenum and jejunum. The improved intestinal morphology was parallel with simultaneously increased intestinal weight by *Lactobacillus* fermentation administration in the present study, in turn suggesting that oral spray with *Lactobacillus* fermentation could stimulate intestinal development to improve nutrients digestion and absorption for eventually better growth performance in ducklings at 21 d of age.

Newly hatched chickens with immature immune function and unstable intestinal flora were susceptible to bacterial infection [9]. Thus, the colonization of beneficial microorganisms should be encouraged to fight against pathogen infection during post-hatch period as soon as possible. An increase amount of *Lactobacillus* was observed in the cecum of ducklings subjected to oral spray with *Lactobacillus* fermentation in the present study. Similar results were observed in newborn birds by feeding [24] or inoculation with *Lactobacillus* strains [27]. Therefore, the improved intestinal microbial environment resulted from *Lactobacillus* fermentation administration of ducklings in the present study. Since *Lactobacillus* administration could increase cecal *Lactobacillus* colonization, *Lactobacillus* could inhibit cecal harmful bacteria colonization by competition for nutrients and adhesion sites on the intestinal epithelium [28]. However, there is no consistent beneficial effects of *Lactobacillus* and metabolites on the antibacterial activity of *E.coli* between Exp. 1 (*in vitro*) and Exp. 2 (*in vivo*). In fact, the degree of *Lactobacillus* effect *in vivo* depends upon the dose or type/blend of *Lactobacillus*, the duration of feeding, bird’s age, overall hygiene conditions on farm and environmental factors. It is implied that the survival ability and adhesive capability of *Lactobacillus* as well as the exact dose and duration of *Lactobacillus* to produce the beneficial effects should be evaluated in our future study. Additionally, *E. coli* colonisations in ducklings reared in a comfortable and clean environment might be kept at a stable and lower level and not be affected easily by *Lactobacillus* treatment.

In conclusion, *Lactobacillus* with the better anti-bacteria ability exhibited a lower antibacterial activity on *E. coli* with antimicrobial resistance than *E. coli* without resistance *in vitro*. *In vivo*, oral spray with *Lactobacillus* fermentation during the first week could improve the intestinal development, morphological structure, and microbial balance to promote growth performance of ducklings from hatch to 21 d of age.

## Figures and Tables

**Table 1:** Composition and nutrient levels of the basal diet (as-fed basis)

Items	
Ingredient (%)	
Corn	56.23
Soybean meal	25.64
Rapeseed meal	8.78
Wheat middling	2.63
Soybean oil	2.53
Dicalcium phosphate	1.42
Limestone	1.09
L-lysine HCl	0.13
DL-methionine	0.20
Salt	0.25
Choline chloride	0.10
Vitamin and mineral premix1)	1.00
Total	100.00
Nutrient levels	Calculated values
Metabolizable energy (MJ/kg)	12.27
Crude protein (%)	20.01
Calcium (%)	0.92
Total phosphorus (%)	0.65
Available phosphorus (%)	0.39
Lysine (%)	1.10
Methionine (%)	0.51
Methionine+cysteine (%)	0.81

1) Provided per kilogram of diet: vitamin A, 4,000 IU; vitamin D_3_, 2,000 IU; vitamin E, 24 IU; thiamine, 2.0 mg; riboflavin, 12 mg; pyridoxine, 4.0 mg; vitamin B_12_, 0.02 mg; calcium pantothenate, 10 mg; folate, 0.15 mg; niacin, 50 mg; biotin, 0.15 mg Choline (Choline chloride), 1,000 mg; Cu (CuSO_4_·5H_2_O), 8 mg; Fe (FeSO_4_·7H_2_O), 80 mg; Zn (ZnSO_4_·7H_2_O), 90 mg; Mn (MnSO_4_·H_2_O), 70 mg; Se (NaSeO_3_), 0.3 mg; I (KI), 0.4 mg.

**Table 2:** Effect of probiotics on the diameter of inhibition zone of *Escherichia coli* without resistance *in vitro*

Inhibition zone	Probiotics groups				p-value
	Saline	*Lactobacillus*	*Bacillus subtilis*	*Lactobacillus*+*Bacillus subtilis*	
Diameter (mm)	-	24.4±0.99^a^	11.1±1.16^c^	19.2±1.30^b^	<0.0001

Data was expressed as mean±standard deviation (n = 3).

“-“, No detected inhibition zone.

a–c Means within the same row lacking a common superscript differ (p<0.05).

**Table 3:** Effect of *Lactobacillus* on the diameter of inhibition zone of *Escherichia coli* with or without resistance *in vitro*

Inhibition zone	*Escherichia coli* sources			p-value
	Saline	Non-resistance	Resistance	
Diameter (mm)	-	24.4±0.50^a^	23.0±1.23^b^	0.0076

Data was expressed as mean±standard deviation (n = 3).

“-“, No detected inhibition zone.

a,b Means within the same row lacking a common superscript differ (p<0.05).

**Table 4:** Effect of oral spray with *Lactobacillus* on growth performance of ducklings from hatch to 21 d of age

Period	Group	Final BW (g/bird)	ADG (g/d/bird)	ADFI (g/d/bird)	F:G (g/g)
D 1–7	Control	159.7±7.7	16.9±1.4	27.6±5.3	1.62±0.20
	Oral spray	165.7±6.0	17.7±1.1	26.5±3.6	1.50±0.18
	p-value	0.26	0.37	0.76	0.39
D 1–21	Control	740.3±20.6^b^	32.5±0.9^b^	66.4±4.1	2.05±0.17
	Oral spray	801.1±37.7^a^	35.3±1.8^a^	70.7±3.8	2.01±0.15
	p-value	0.03	0.03	0.17	0.72

Data was expressed as mean±standard deviation (n = 4).

BW, body weight; ADG, average daily gain; ADFI, average daily feed intake; F:G, feed:gain ratio.

a,b Means within the same column lacking a common superscript differ (p<0.05).

**Table 5:** Effect of oral spray with *Lactobacillus* on the absolute intestinal weight and length of ducklings at 21 d of age

Items	Group	Duodenum	Jejunum	Ileum	Total tract
Absolute weight (g)	Control	4.45±0.53	10.0±0.89^b^	9.25±0.75^b^	23.7±1.7^b^
	Oral spray	4.84±0.68	11.3±1.20^a^	10.7±1.38^a^	26.9±2.7^a^
	p-value	0.22	0.02	0.02	0.01
Absolute length (cm)	Control	26.3±2.0	62.8±4.6	59.5±4.4	148.6±9.4
	Oral spray	26.8±2.0	64.7±4.0	64.3±7.2	155.8±10
	p-value	0.63	0.39	0.13	0.16

Data was expressed as mean±standard deviation (n = 8).

a,b Means within the same column lacking a common superscript differ (p<0.05).

**Table 6:** Effect of oral spray with *Lactobacillus* on intestinal histomorphology of ducklings at 21 d of age

Segments	Group	Villus height (μm)	Crypt depth (μm)	Musculature thicknesses (μm)	Villus height:crypt depth
Duodenum	Control	365±134^b^	144±23^b^	542±136	2.49±0.64^b^
	Oral spray	436±89^a^	123±30^a^	585±74	3.84±1.43^a^
	p-value	0.02	0.001	0.14	<0.0001
Jejunum	Control	296±89^a^	111±21	438±115^b^	2.76±0.99^b^
	Oral spray	396±80^b^	107±34	617±91^a^	4.08±1.68^a^
	p-value	<0.0001	0.59	<0.0001	<0.0001
Ileum	Control	325±45	102±16	446±50	3.28±0.74
	Oral spray	343±67	112±23	475±85	3.10±0.38
	p-value	0.24	0.07	0.11	0.24

Data was expressed as mean±standard deviation (n = 8).

a,b Means within the same column lacking a common superscript differ (p<0.05).

**Table 7:** Effect of oral spray with *Lactobacillus* on cecum flora amount of ducklings at 21 d of age (log CFU/g)

Items	*Escherichia coli*	*Lactobacillus*	Total colonies	*Lactobacillus:Escherichia coli* ratio
Control	8.65±0.83	8.30±0.97^b^	7.86±0.58	0.97±0.18
Oral spray	8.01±0.46	9.59±0.15^a^	8.46±0.19	1.20±0.08
p-value	0.22	0.038	0.09	0.06

CFU, colony forming units.

Data was expressed as mean±standard deviation (n = 4).

a,b Means within the same column lacking a common superscript differ (p<0.05).
